# Vaccination Management and Vaccination Errors: A Representative Online-Survey among Primary Care Physicians

**DOI:** 10.1371/journal.pone.0105119

**Published:** 2014-08-13

**Authors:** Birgitta M. Weltermann, Marta Markic, Anika Thielmann, Stefan Gesenhues, Martin Hermann

**Affiliations:** Institute for General Medicine, Essen University Hospital, University of Duisburg-Essen, Essen, Germany; Universidad Nacional de La Plata, Argentina

## Abstract

**Background:**

Effective immunizations require a thorough, multi-step process, yet few studies comprehensively addressed issues around vaccination management.

**Objectives:**

To assess variations in vaccination management and vaccination errors in primary care.

**Methods:**

A cross sectional, web-based questionnaire survey was performed among 1157 primary physicians from North Rhine-Westphalia, Germany: a representative 10% random sample of general practitioners (n = 946) and all teaching physicians from the University Duisburg-Essen (n = 211). Four quality aspects with three items each were included: patient-related quality (patient information, patient consent, strategies to increase immunization rates), vaccine-related quality (practice vaccine spectrum, vaccine pre-selection, vaccination documentation), personnel-related quality (recommendation of vaccinations, vaccine application, personnel qualification) and storage-related quality (storage device, temperature log, vaccine storage control). For each of the four quality aspects, “good quality” was reached if all three criteria per quality aspect were fulfilled. Good vaccination management was defined as fulfilling all twelve items. Additionally, physicians’ experiences with errors and nearby-errors in vaccination management were obtained.

**Results:**

More than 20% of the physicians participated in the survey. Good vaccination management was reached by 19% of the practices. Patient-related quality was good in 69% of the practices, vaccine-related quality in 73%, personnel-related quality in 59% and storage-related quality in 41% of the practices. No predictors for error reporting and good vaccination management were identified.

**Conclusions:**

We identified good results for vaccine- and patient-related quality but need to improve issues that revolve around vaccine storage.

## Introduction

Effective immunizations require a thorough, multi-step process on behalf of the physicians and the practice teams in charge. In many Western countries, detailed recommendations address not only the medical indications for the various vaccinations, but also structural and procedural aspects of the vaccination management. Although millions of vaccinations are performed each year [1], relatively few studies address the every-day challenges of practices’ vaccination management. Unsystematic observations in our region suggest marked differences between practices with regard to patient information, how to obtain patient consent, the involvement of practice assistants in vaccinations and the vaccine handling. Aiming to understand variations in vaccination management in primary care, we performed a web-based questionnaire survey among primary care physicians in Germany’s largest federal state North Rhine-Westphalia.

## Methods

### Ethics statement

Ethical approval was obtained from the ethic commission of the medical faculty of the University of Duisburg-Essen.

### Survey design and study populations

We performed a cross-sectional, web-based questionnaire survey among a random and a convenience sample of primary care physicians in North Rhine-Westphalia, Germany’s largest federal state. The survey included general practitioners (with and without board certification) and general internists, paediatricians were excluded. Primary care physicians in North Rhine-Westphalia are organized in the two regional Statutory Health Care Administrations North Rhine and Westphalia-Lippe. We draw a 10% random sample with a total of 946 of 9533 primary care physicians: 576 of 5757 physicians in North Rhine and 378 of 3776 physicians in Westphalia-Lippe. In addition, all primary care teaching physicians of the University of Duisburg-Essen, Essen (n = 211), were invited to participate in the survey.

### Survey instrument

The online questionnaire survey was constructed using LimeSurvey (www.limesurvey.org). The questionnaire consisted of 30 questions addressing immunization aspects relevant for daily primary care. The items were developed and selected based on three practice visits and a systematic comparison of four national guidelines (United Kingdom [2], United States of America [3], [4], Australia [5], [6], Germany [7]–[10]). The first part of the questionnaire addressed issues of vaccination management, the second part errors and near-errors while the third part requested social demographic data. The questionnaire was pre-tested on the research team and three external academic primary care physicians.

#### Quality indicators

For the first part of the questionnaire a modified Delphi technique with five primary care teaching physicians was used to identify relevant quality aspects. The four selected items were: patient-related quality, vaccine-related quality, personnel-related and storage-related quality. In each of these four categories, three items were selected as relevant quality indicators:

- *Patient-related quality*:
  Patient information: in four case studies physicians provided all patients with information
  Patient consent: consensus obtained always verbally and/or written
  Three strategies to increase immunization rates were used: the physician often or always controls the immunization status of new patients, patients in risk groups and provides patients with a follow-up appointment for the next vaccination
- *Vaccine-related quality*:
  Practice vaccination spectrum: all standard vaccinations (tetanus, diphtheria, poliomyelitis, pneumococcal disease if age ≥60, influenza if age ≥60) are offered. Note: when the survey was designed in July 2009, pertussis was newly introduced as standard vaccination for adults. This recent change was not included in the definition of the quality indicator
  Vaccine pre-selection: a person is designated as responsible to preselect vaccines
  Chart documentation of vaccinations: charge number, dose, trade name (Note: in Germany these items need to be documented separately in addition to billing data which contain the responsible physician, date and disease against which is vaccinated)
- *Personnel-related quality:*
  Vaccine indications: in one clinical vignette the relevant vaccines according to guidelines are chosen
  Vaccine application: designated personnel applies vaccines
  Personnel qualification: the physician and/or medical assistant participated in a vaccination-related CME within the last two years
- Storage-related quality:
  Storage device: a separate refrigerator is used
  Temperature: keeping a storage temperature log
  Regular vaccine storage control: with regard to the criteria wrapping, expiration date and temperature

For each of the four quality aspects, “good quality” was reached if all three criteria per quality aspect were fulfilled. Reaching all four quality indicators was called “good vaccination management”.

### Errors and near-errors in vaccination management

In the second part of the questionnaire, physicians’ experiences with 15 vaccination errors were addressed. Items were based on the anonymous German critical incident reporting system “Jeder Fehler Zaehlt” (ie, “every error counts”; www.jeder-fehler-zaehlt.de). Answer options for each item were: not experienced, experienced as near-error or experienced as actual error.

### Data collection

The survey was hosted on the LimeSurvey platform. Participants were invited by email or - if email address was missing - letter and provided with login information. Participants were asked to complete the anonymous questionnaire within six weeks and received a reminder after three weeks.

### Statistical analysis

Analyses were conducted using SPSS Statistics, version 20. For the descriptive statistics, the frequencies of various answers were calculated. The quality of vaccination management was assessed using one quality indicator each for patient-, vaccine- and personnel and storage-related aspects. Each indicator consisted of the above mentioned three items and scoring positive on all these served as cut-off point for good quality. Also, a summarizing quality indicator called “good vaccination management” was developed through the summation of scores above the cut-off points on the four indicators; scoring positive on all 12 indicators was considered good quality. Chi-square tests were used to analyse if independent parameters (practice and physician characteristics) influenced the frequencies of scoring on the four quality indicators and the overall quality indicator for good vaccination management. A statistical significance was assigned at a level of p<0.05. Logistic regression analysis was used to determine predictors for good vaccination management. Factors associated with good vaccination management quality in bivariate analysis were included in the final model.

## Results

### Study participation

A total of 247 of 1157 physicians participated in the survey (response rate: 21%). The sub-analyses showed that 127 of 211 teaching physicians and 120 of 946 physicians in the random sample participated. The questionnaire was completed by 74% of the random sample and 65% of the university sample. Formal comparison showed that completion of the questionnaire was positively correlated with a larger vaccine spectrum in the random sample (Spearman r_s_ = 0.191, p = 0.037), in the teaching sample no correlation was identified. Analyses included completed questionnaires only (n = 172). The two samples showed no significant differences in their structural characteristics except that the teaching practice sample showed more practices with two or more physicians (65% vs. 46%, p<0.01), more practices with more than 1500 patients (59% vs. 34%, p<0.01) and more physicians with palliative care training (29 vs. 16%, p = 0.04). For details see Table 1 and Table S1.

**Table 1:** Physician and practice characteristics of survey respondents.

	Study population	
	N	%
Surveyed	1157	
No. of respondents (rate)	247	21
**Questionnaires in final analysis** #	172	
**Physician characteristics**		
*Mean age (range)*	51	(39–67)
*Males*	126	73
*Degree*		
GP (b.c.)	109	63
General internal medicine (b.c.)	51	30
Practitioner without degree (b.e.)	8	5
*Additional qualifications* *		
Travel medicine	35	20
Complementary medicine	37	22
Palliative care	38	22
Other (*ε*26)	68	40
*Physician vaccination within last 2 yrs*	129	75
*Family member vaccination within last 2 yrs*	152	88
**Practice characteristics**		
*Practice setting*		
Solo	75	44
Group/2-person	58	34
Group/≥3-person	37	22
*Has physicians in training*	30	17
*Academic affiliation*	101	59
*No. of patients in practice (quarterly)*		
≤1000	37	22
1001–1500	54	31
≥1501	79	46
*Team member vaccination within last 2 yrs*	159	92

*Multiple response.

# The final analysis included completed questionnaires only.

b.c.: board certified.

b.e.: board eligible.

### Physician and practice characteristics

The majority of the physicians was male with an average age of 51 years. 63% were GPs, 30% held a specialty degree in general internal medicine, and 5% were practitioners without degree. Almost half of the physicians (44%) were working in a solo practice. The majority (77%) had practices with more than 1000 patients quarterly. For details see Table 1 and Table S1.

### Vaccination management

The indicators were met as follows: 69% met the patient-related quality indicator, 73% the vaccine-related quality indicator, 59% the personnel-related quality indicator and 41% the storage-related quality indicator. The overall quality indicator for good vaccination management was reached by 19%. Comparison of the quality indicators in the two samples showed no significant difference except that the percentage of practices fulfilling the patient consent quality indicator was 96% in the random sample and 87% among the teaching physicians (p = 0.02). For details see Table S2. Potential for improvement was documented for all storage-related aspects and single other items: only 79% used a separate refrigerator for vaccine storage, 51% kept a storage temperature log, 92% performed a regular storage control (wrapping, temperature and expiration date), 77% had a complete chart documentation (86% the charge number and dose, 84% the trade name), 66% recommended vaccinations according to current recommendations. Practice strategies to increase vaccination coverage were used often or routinely by 76% of practices. For details see Table 2.

**Table 2:** Quality indicators for vaccination management.

	Study population	
	(n = 172)	
	n	%
**Patient-related quality**		
Patient information (any; always)	169	98
Patient consent (always written and/or verbal)	157	91
Strategies to increase immunization rates	130	76
**Quality Indicator Patient (3/3)**	119	69
**Vaccine-related quality**		
Spectrum of standard vaccines used in practice*	163	95
Designated person for pre-selection	171	99
Chart documentation (charge number/dose, trade name)	132	77
**Quality Indicator Vaccine (3/3)**	126	73
**Personnel-related quality**		
Correct vaccination recommendations	114	66
Physician or designated personnel applies vaccines	170	99
CME≤2 yrs (physician and/or assistant)	147	86
**Quality Indicator Personnel (3/3)**	102	59
**Storage-related quality**		
Separate refrigerator	136	79
Storage temperature log	87	51
Regular storage control (wrapping, temperature, expiration date)	158	92
**Quality indicator Storage (3/3)**	70	41
**Vaccination Management Quality Indicator (12/12)**	32	19

*Standard vaccinations were defined as pneumococci, influenza, diphtheria, poliomyelitis and tetanus.

Various additional characteristics of practice vaccination management not included in the quality indicators were obtained. Of the 20 vaccines offered, the practices used on average 16.6 vaccines (8–20, SD: 2.28), 95% offered all of the five standard vaccinations, 6% offered all 15 indication vaccinations (on average 11.7 vaccines). The following pattern of responsibilities within the practice team was identified: in most practices physicians were responsible for the vaccine pre-selection (79%) and providing patient information (83%), whereas in the majority of practices medical assistants were designated as solely responsible for vaccine ordering (73%), stock control (82%) and storage control (60%). All practices (99%) reported that either the physician or the medical assistant is responsible for the storage control. In 20% of the practices vaccines were administered by the physician only, while the decision who inoculates was case-specific in 56% of the practices, depending either on the vaccine or the patient. The participation rate in vaccination-related CME within the last two years was 77% in physicians and 70% in medical assistants. A recall to all patients was offered by 41% of practices. Regular practice software for the development of patients’ lists was used by 35%, additional immunization specific software by 4%. Results on additional characteristics of practice vaccination management are presented in Table 3.

**Table 3:** Additional characteristics of vaccination management.

	Study population	
	(n = 172)	
	n	%
**Practice vaccine spectrum**		
Mean no. of vaccines (range)	16.6 (8–20)	
*Standard vaccines*		
Influenza	172	100
Pneumococcal disease	170	99
Poliomyelitis	170	99
Diphtheria	169	98
Tetanus	169	98
*Indication vaccines*		
Hepatitis A	172	100
Tick-borne encephalitis	172	100
Hepatitis B	171	99
Pertussis	169	98
Rubella	165	96
Measles	163	95
Mumps	162	94
Meningococcal disease	149	87
Typhus	140	81
Human papilloma	123	72
Rabies	120	70
Varicella	120	70
Haemophilus influenzae b	99	58
Cholera	72	42
Yellow fever (restricted license required)	19	11
**Responsibilities within practice team**		
Vaccine pre-selection, physician only	136	79
Ordering, medical assistant only	126	73
Storage control, medical assistant only	103	60
Stock control, medical assistant only	141	82
Patient information, physician only	143	83
Vaccine application by physician only	35	20
Administrator depends on vaccination*	97	56
Personnel inoculating depends on patient*	98	57
**Vaccine pre-selection criteria** *		
Easy handling	88	51
Cheapest price	55	32
Specific company	36	21
**Strategies to increase immunization rates**		
Control of each new patient	148	86
Appointment for next vaccination	154	90
Control of risk groups	163	95
During consultation	155	90
Telephone	53	31
General formulated letter	24	14
Written patient notification	23	13
Sms or e-mail	13	8

*Multiple response.

### Error and near-error reporting

At least one error or near-error was reported by 89% of the participants. On average, a total of 3.4 errors and near-errors had occurred: 2.3 errors and 1.2 near-errors. The five most frequently reported types of errors were: double vaccination due to missing documentation in 40%, intramuscular injection of a patient on anticoagulants therapy in 39%, vaccination despite missing indication in 36%, wrong vaccine in 19%, and the vaccination of a 14-year-old without parental approval in 16%. The three most frequently reported near-errors were: vaccination despite of acute disease in 13%, intramuscular injection of a patient on anticoagulants therapy in 13%, and expired vaccine in 12%. For details on the sum of all 15 errors and near-errors see Table 4 and Table S3.

**Table 4:** Frequencies of errors and near-errors in vaccination management*.

	Study population	
	(n = 172)	
Type of error/near-error	n	%
Intramuscular injection of patient on anticoagulants	89	52
Double vaccination due to lack of documentation	84	49
Vaccination without indication	75	44
Wrong vaccine	52	30
Expired vaccine	42	24
Vaccinated despite acute disease	37	22
14-year-old vaccinated without parental approval	38	22
Wrong vaccine dose	32	19
Wrong travel vaccination recommended	31	18
Wrong temperature in refrigerator	27	16
Wrong vaccination administration	28	16
Staff vaccinated without physician’s order	22	13
Wrong patient inoculated	17	10
Reminder send to patient with new family doctor	16	9
A pregnant woman receives rubella inoculation	0	0

*The items offered were based on reports in a German primary care incidents reporting system.

### Physicians’ opinions about the survey

The survey was considered helpful by 30%, triggered a reconsideration of practice standards in 38% and caused the wish to participate in CME in 16%. Taking immediate action was reported by 10% of physicians, 69% voiced an interest in the results.

### Predictors for good vaccination management quality and error reporting

One fifth (19%) of all practices showed “good vaccination management”. Chi-square tests for the four quality indicators and the overall quality indicator for good vaccination management did not identify any parameters influencing all quality indicators. Chi-square tests did not reveal any parameters influencing error reporting. The following predictors for good vaccination management quality were identified:

- *Quality Indicator Storage:* additional qualification in travel medicine (60% vs. 36%, p = 0.010, x^2^ = 6.615)
- *Quality Indicator Personnel:* group practice (71% vs. 45%, p = 0.001, x^2^ = 11.031), additional qualification in travel medicine (80% vs. 54%, p = 0.006, x^2^ = 7.572), practices with more than 1500 patients quarterly (70% vs. 51%, p = 0.012, x^2^ = 6.378)

Logistic regression did not identify any predictors for good vaccination management.

## Discussion

To our knowledge this is the first comprehensive study among primary care physicians about vaccination management: using twelve quality indicators we showed that 19% of practices had good practice vaccination management, while a marked variance between practices and the need to improve storage conditions became apparent.

### Patient-related process quality

In the medical literature, three groups of interventions were studied as strategies to increase immunization rates in children and adults: patient-oriented interventions (e.g. written reminders), provider interventions (e.g. preventive services flow sheets in patient charts) and system interventions (e.g. public immunization campaigns) [11]. A Cochrane review comparing RCTs with patient-oriented interventions showed that postcards, letters, autodialers, and person-to-patient phone calls increased immunization rates: phone reminders were most effective but also most costly, yet increased immunization rates up to thirty percent [11]. In 2000, a survey among 316 US primary care physicians showed that 23% of practices are using mail or phone reminders [12]. This is comparable to our results which showed that 31% of practices are using phone, and up to 14% written reminders, e.g. sms, e-mail, or letter. Provider interventions were more frequent than patient-oriented interventions both in the US survey and our study, yet the measures used differ. In decreasing order, US physicians used the following three strategies most frequently: preventive service flow sheets in patient charts (71%), walk-in immunization service (67%), and a policy to assess vaccination status at each visit [12]. In comparison, our survey showed that physicians apply the strategy to control risk groups (95%), provide appointment for next vaccination (90%) and use routine consultations to optimize immunization levels (90%). These differences may be due to health care system factors, physician education, and individual preferences. System differences also play a role with regard to the mode of patient consent and information: while verbal information and verbal patient consent is sufficient by German law, US law requires the provision of detailed written consent prior to each vaccination [4].

### Vaccine-related process quality

Adequately trained personnel is of key importance to assure a high quality of vaccine management. This was documented in an intervention study in the Atlanta region: daily temperature monitoring of vaccine storage compartments was 2–3 times more likely if the designated coordinator had a higher level of medical education [13]. A survey of 221 US practices showed that 83% had designated a specific person as responsible for vaccine storage and handling, with a backup in 63% of practices [13]. Another US survey among 721 primary care offices differentiated between ordering, storing and application of vaccines: in the majority of practices only one person was responsible for ordering vaccines (75%), two or more staff members were responsible for storing (50%) and application of vaccines (77%) [14]. Our survey analyzed responsibilities within the practice team: in more than 70% of practices it is the sole responsibility of the physician in charge to pre-select vaccines, while ordering, storage and stock control are typically delegated to medical assistants. We consider it positive that physicians are involved in vaccine pre-selection, as these decisions may pre­vent programmatic vaccination errors described in the medical literature, such as mixed use of brands for the same vaccination or the use of different vaccines with similar names [15]–[17].

### Personnel-related quality

The definition of our quality indicator addressed three typical aspects of personnel-related immunization quality: application of vaccination recommendations, personnel qualification and the physician or a designated person applies the vaccine. Considering the recommendation of catch-up vaccination regimes, studies among pediatricians showed a broad variance between physicians [18]–[20], which was confirmed in our study. Using one clinical vignette we showed discrepancies to current recommendations. These differences may be oblivious due to knowledge deficits (e.g. with regard to immunization recommendations and contraindications), or deliberate due to disagreement with immunization recommendations. Both aspects have been previously associated with the omission of vaccinations, especially in pediatricians [12], [19]. Discussed are also increasingly complex immunization schedules as barrier to immunization practices conform to recommendations [20], [19].

### Storage-related quality

The definition of our quality indicator addressed three typical storage-related aspects: storage device, keeping a storage temperature log and regular storage control. In our study, 79% of practices used a separate refrigerator for vaccines, which was documented in 96% of 695 US primary care practices [14], yet only 59% of 172 Australian [21], and 9 to 22% of 135 Canadian practices [22]. All of the practices surveyed in our study had designated coordinators responsible for storage control, which is higher compared to 83% US private providers who designated a person for the joint tasks vaccine storage and handling [13]. Frequently, vaccine storage conditions are a weak point in the quality chain with the potential to cause disease outbreaks [23]–[25], to reduce the vaccine effectiveness and the tolerability of vaccines [26], [27]. A systematic literature review based on 14 studies in developed countries showed that 13.5% (6.4 to 20.7%) of refrigerators had temperatures below the freeze threshold [28]. The difficulty of maintaining refrigerators within the correct temperature range becomes clear considering that even after participating in an intervention study, 50% of the practices fail this criteria [29]. Additionally, the storage equipment used is crucial. An Australian study based on 28 general practitioners showed that the refrigerator type used is associated with maintaining correct temperatures, with purpose built vaccine refrigerators showing better results [30]. Our results are in agreement with these findings: only 92% regularly controlled their storage and only 51% of the practices kept a storage temperature log. The latter result is much lower compared to 73% US primary care practices [14], yet comparable to 53% US private physicians who do not have a log [13]. Thus, at least in our region, future interventions to improve vaccine-related quality should address the issue of a separate refrigerator, a storage temperature log and regular storage control as important content of CME and practice improvement strategies.

### Errors and near-errors

In our understanding, the reported average of 3.4 errors and near-errors indicates good error awareness. In accordance with Bundy et al. who analyzed 607 error reports on an US error reporting database (“MEDMARX”) [17] we can confirm high rates for those types of errors reported most frequently in that database: double vaccination (40% vs. 25%) and wrong vaccine (19% vs. 25%). Thus, interventions should focus e.g. on improving vaccination documentation to reduce doubled vaccination (e.g. using electronic health records) and prevent look-alike/sound-alike confusion to reduce using the wrong vaccine.

### Limitations, Conclusions and Perspectives

Our cross-sectional physician survey addressed a broad spectrum of issues. Several limitations need to be considered. First, it was performed in a country lacking detailed guidelines on vaccination management and the quality indicators used are based on common reasoning and standards from other countries. Second, we did not compare physicians’ answers with actual every-day vaccination management. Third, given the low response rate, a response bias and a bias towards the desired answers cannot be excluded, e.g. regarding time since last CME.

In summary, we demonstrated that about 19% of German primary care practices have good vaccination management, yet broad variance for single aspects. Interventions should focus on storage-related issues.

## Supporting Information

Table S1 **Comparison of physician random sample and teaching physicians for physician and practice characteristics.**
(DOCX)

Table S2 **Comparison of physician random sample and teaching physicians for quality indicators for vaccination management.**
(DOCX)

Table S3 **Comparison of physician random sample and teaching physicians for frequencies of errors and near-errors in vaccination management.**
(DOCX)
