# Supplementary material for: Vaccination Management and Vaccination Errors: A Representative Online-Survey among Primary Care Physicians
Source: PLoS One. 2014 Aug 13;9(8):e105119. doi: 10.1371/journal.pone.0105119 (PMC4132103; doi:10.1371/journal.pone.0105119)
Supplement: Table S1 — Comparison of physician random sample and teaching physicians for physician and practice characteristics. (DOCX) [file pone.0105119.s001.docx]

**Supplement 1**

***Table S1: Comparison of physician random sample and teaching physicians for physician and practice characteristics***

|  | Total Population | | Random Sample | | Teaching Physicians | | P-value** |
| --- | --- | --- | --- | --- | --- | --- | --- |
|  | N | % | N | % | N | % |  |
| Surveyed | 1157 |  | 954 |  | 211 |  |  |
| No. of respondents (rate) | 247 | 21 | 120 | 13 | 127 | 60 |  |
| **Questionnaires in final analysis#** | 172 |  | 89 |  | 83 |  |  |
| **Physician characteristics** |  |  |  |  |  |  |  |
| *Mean age (range)* | 51 | (39-67) | 52 | (39-67) | 50 | (39-64) | n.s. |
| *Males* | 126 | 73 | 63 | 71 | 63 | 76 | n.s. |
| *Degree* |  |  |  |  |  |  |  |
| GP (b.c.) | 109 | 63 | 51 | 57 | 58 | 70 | n.s. |
| General internal medicine (b.c.) | 51 | 30 | 27 | 30 | 24 | 29 | n.s. |
| Practitioner without degree (b.e.) | 8 | 5 | 7 | 8 | 1 | 1 | n.s. |
| *Additional qualifications** |  |  |  |  |  |  |  |
| Travel medicine | 35 | 20 | 17 | 19 | 18 | 22 | n.s. |
| Complementary medicine | 37 | 22 | 15 | 17 | 22 | 27 | n.s. |
| Palliative care | 38 | 22 | 14 | 16 | 24 | 29 | 0.04 |
| Other ($\varepsilon26)$ | 68 | 40 | 32 | 36 | 36 | 43 | n.s. |
| *Physician vaccination within last 2 yrs* | 129 | 75 | 65 | 73 | 64 | 77 | n.s. |
| *Family member vaccination within last 2 yrs* | 152 | 88 | 79 | 89 | 73 | 88 | n.s. |
| **Practice characteristics** |  |  |  |  |  |  |  |
| *Practice setting* |  |  |  |  |  |  |  |
| Solo | 75 | 44 | 47 | 53 | 28 | 34 | 0.01 |
| Group/2-person | 58 | 34 | 24 | 27 | 34 | 41 | n.s. |
| Group/≥3-person | 37 | 22 | 17 | 19 | 20 | 24 | n.s. |
| *Has physicians in training* | 30 | 17 | 10 | 11 | 20 | 24 | n.s. |
| *Academic affiliation* | 101 | 59 | 18 | 20 | 83 | 100 | 0.00 |
| *No. of patients in practice (quarterly)* | |  |  |  |  |  |  |
| ≤ 1000 | 37 | 22 | 26 | 29 | 11 | 13 | 0.01 |
| 1001-1500 | 54 | 31 | 32 | 36 | 22 | 27 | n.s. |
| ≥ 1501 | 79 | 46 | 30 | 34 | 49 | 59 | 0.00 |
| *Team member vaccination within last2 yrs* | 159 | 92 | 82 | 92 | 77 | 93 | n.s. |

* Multiple response

** Continuous variables were tested using the t-test, categorical variables using chi-square-test.

# The final analysis included completed questionnaires only

b.c.: board certified

b.e.: board eligible
