# Supplementary material for: Vaccination Management and Vaccination Errors: A Representative Online-Survey among Primary Care Physicians
Source: PLoS One. 2014 Aug 13;9(8):e105119. doi: 10.1371/journal.pone.0105119 (PMC4132103; doi:10.1371/journal.pone.0105119)
Supplement: Table S2 — Comparison of physician random sample and teaching physicians for quality indicators for vaccination management. (DOCX) [file pone.0105119.s002.docx]

**Supplement 2**

***Table S2: Comparison of physician random sample and teaching physicians for quality indicators for vaccination management***

|  | Total Population | | Random Sample | | Teaching Physicians | | P-value |
| --- | --- | --- | --- | --- | --- | --- | --- |
|  | (n=172) | | (n=89) | | (n=83) | |  |
|  | n | % | n | % | n | % |  |
| **Patient-related quality** |  |  |  |  |  |  |  |
| Patient information (any; always) | 169 | 98 | 87 | 98 | 82 | 99 | n.s. |
| Patient consent (always written and/or verbal) | 157 | 91 | 85 | 96 | 72 | 87 | 0.02 |
| Strategies to increase immunization rates | 130 | 76 | 70 | 79 | 60 | 72 | n.s. |
| **Quality Indicator Patient (3/3)** | 119 | 69 | 66 | 74 | 53 | 64 | n.s. |
| **Vaccine-related quality** |  |  |  |  |  |  |  |
| Spectrum of standard vaccines used in practice* | 163 | 95 | 84 | 94 | 79 | 95 | n.s. |
| Designated person for pre-selection | 171 | 99 | 88 | 99 | 83 | 100 | n.s. |
| Chart documentation (charge number/dose, trade name) | 132 | 77 | 70 | 79 | 62 | 75 | n.s. |
| **Quality Indicator Vaccine (3/3)** | 126 | 73 | 67 | 75 | 59 | 71 | n.s. |
| **Personnel-related quality** |  |  |  |  |  |  |  |
| Correct vaccination recommendations | 114 | 66 | 60 | 67 | 54 | 65 | n.s. |
| Physician or designated personnel applies vaccines | 170 | 99 | 87 | 98 | 83 | 100 | n.s. |
| CME ≤ 2 yrs (physician and/or assistant) | 147 | 85 | 76 | 85 | 71 | 86 | n.s. |
| **Quality Indicator Personnel (3/3)** | 102 | 59 | 53 | 60 | 49 | 59 | n.s. |
| **Storage-related quality** |  |  |  |  |  |  |  |
| Separate refrigerator | 136 | 79 | 67 | 75 | 69 | 83 | n.s. |
| Storage temperature log | 87 | 51 | 43 | 48 | 44 | 53 | n.s. |
| Regular storage control (wrapping, temperature, expiration date) | 158 | 92 | 79 | 89 | 79 | 95 | n.s. |
| **Quality indicator Storage (3/3)** | 70 | 41 | 35 | 39 | 35 | 42 | n.s. |
| **Vaccination Management Quality Indicator (12/12)** | 32 | 19 | 15 | 17 | 17 | 21 | n.s. |

* Standard vaccinations were defined as pneumococci, influenza, diphtheria, poliomyelitis and tetanus
