# Supplementary material for: Vaccination Management and Vaccination Errors: A Representative Online-Survey among Primary Care Physicians
Source: PLoS One. 2014 Aug 13;9(8):e105119. doi: 10.1371/journal.pone.0105119 (PMC4132103; doi:10.1371/journal.pone.0105119)
Supplement: Table S3 — Comparison of physician random sample and teaching physicians for frequencies of errors and near-errors in vaccination management. (DOCX) [file pone.0105119.s003.docx]

**Supplement 3**

***Table S3: Comparison of physician random sample and teaching physicians for frequencies of errors and near-errors in vaccination management****

|  | Total Population | |  | Random Sample | |  | Teaching Physicians | |  | P-value |
| --- | --- | --- | --- | --- | --- | --- | --- | --- | --- | --- |
|  | (n=172) | |  | (n=89) | |  | (n=83) | |  |  |
| **Type of error / near-error** | n | % |  | n | % |  | n | % |  |  |
| Intramuscular injection of patient on anticoagulants | 89 | 52 |  | 42 | 47 |  | 47 | 57 |  | n.s. |
| Double vaccination due to lack of documentation | 84 | 49 |  | 47 | 53 |  | 37 | 45 |  | n.s. |
| Vaccination without indication | 75 | 44 |  | 37 | 42 |  | 38 | 46 |  | n.s. |
| Wrong vaccine | 52 | 30 |  | 25 | 28 |  | 27 | 33 |  | n.s. |
| Expired vaccine | 42 | 24 |  | 20 | 23 |  | 22 | 27 |  | n.s. |
| Vaccinated despite acute disease | 37 | 22 |  | 19 | 21 |  | 18 | 22 |  | n.s. |
| 14-year-old vaccinated without parental approval | 38 | 22 |  | 17 | 19 |  | 21 | 25 |  | n.s. |
| Wrong vaccine dose | 32 | 19 |  | 17 | 19 |  | 15 | 18 |  | n.s. |
| Wrong travel vaccination recommended | 31 | 18 |  | 20 | 23 |  | 11 | 13 |  | n.s. |
| Wrong temperature in refrigerator | 27 | 16 |  | 19 | 21 |  | 8 | 10 |  | 0.03 |
| Wrong vaccination administration | 28 | 16 |  | 15 | 17 |  | 13 | 16 |  | n.s. |
| Staff vaccinated without physician’s order | 22 | 13 |  | 11 | 12 |  | 11 | 13 |  | n.s. |
| Wrong patient inoculated | 17 | 10 |  | 12 | 14 |  | 5 | 6 |  | n.s. |
| Reminder send to patient with new family doctor | 16 | 9 |  | 7 | 8 |  | 9 | 11 |  | n.s. |
| A pregnant woman receives rubella inoculation | 0 | 0 |  | 0 | 0 |  | 0 | 0 |  | n.s. |

******* The items offered were based on reports in a German primary care incidents reporting system.
